# Correlates of preschoolers’ screen time in China: parental factors

**DOI:** 10.1186/s12887-022-03443-7

**Published:** 2022-07-14

**Authors:** Xinyao Wang, Yan Wu, Chunhua Yao, Xiangting Wu, Yuqian Ruan, Sunyue Ye

**Affiliations:** 1 Department of Preschool Education, Pinghu Normal College, Jiaxing University, No. 888, Hongjian Road, Pinghu, 314001 Jiaxing, China; 2 Pinghu Kindergarten in Economic Development Zone, Jiaxing, 314201 China

**Keywords:** Preschooler, Screen exposure, Sedentary behavior, Early development, Risk factors

## Abstract

**Background:**

With the advent of the electronic age, the prolonged screen time (ST) of preschoolers in China is relatively high and is on the rise, which is likely to affect preschoolers’ physical and mental health. This study aimed to explore the factors influencing ST in preschoolers, especially the role of parental factors, and to provide a basis for the prevention, control, and intervention of ST in preschoolers in China.

**Methods:**

A questionnaire was completed by the parents of 1,546 preschoolers from four kindergartens in Pinghu City, Zhejiang Province, China, and a multivariate logistic regression model was used to analyze the correlates of excessive ST in preschoolers.

**Results:**

A total of 43.8% of preschoolers aged 3 to 6 years, of which 50.3% were boys and 49.7% were girls, had > 1 h/day of ST. Older preschoolers, greater screen accessibility, greater frequency of eating in front of a screen, longer ST of parents, and unclear rules of screen-based behavior were the risk factors for ST being > 1 h/day (*P* < 0.05). After additional adjusting of maternal correlates, the relationship between the ST of fathers and ST of preschoolers was still significant (*P* < 0.01), and the dose–effect relationship was also observed (*P* < 0.001).

**Conclusion:**

Prolonged parental ST (especially of fathers) and lack of rules for screen behavior were independent risk factors for prolonged preschoolers’ ST in this study.

## Background

Screen time (ST) refers to the time spent doing sedentary activities in front of a screen, for example, watching television, using a computer, and playing on smartphones or electronic games [1], and is closely related to many health problems such as myopia and obesity [2, 3]. Preschoolers, known as the “electronic media generation”, grow up in families and social environments surrounded by various screens. There is a trend of children using electronic media for the first time at younger ages, with long-term screen exposure being common in preschoolers at school and at home [4]. According to previous studies, the prevalence of ST exceeding guidelines of preschoolers in China is relatively high and shows a rising trend [5, 6]. The average ST of children aged three to six is greater then 1 h/day, and on weekends is > 2 h/day [7]. At the same time, the overall prevalence of myopia in children and adolescents in China remains high (57.2%) and shows a trend of affecting children at a younger age. This may be closely related to exposure to near-sighted environments, such as excessive ST [8]. The health behavior-related guidelines issued by the World Health Organization, developed countries in Europe, the United States, and relevant institutions in China all recommend that the ST of preschoolers be restricted (for example, to no more than 1 h/day and as few hours as possible) [9].

Parental education levels, parental screen use or ST, accessibility of electronic devices, parental screen-based behaviors rules and their attitudes toward children’ screen use have been found to be associated with ST in preschoolers, children, or adolescents [10–13]. These indicated that parental factors seems to be a essential for cultivating screen-based behaviors in preschoolers. However, existing literature on factors influencing preschoolers’ ST is still limited, further exploring is needed, especially for developing countries such as China. Therefore, this study aimed to explore the factors affecting the home-based ST of preschoolers, and especially the influence of parents, in order to prevent and control home-based ST effectively in Chinese preschoolers.

## Methods

### Participants

From September to October 2019, the parents of preschoolers aged 3 to 6 years old (*n* = 1,546) from four kindergartens in Pinghu, China, participated in this study, the retain rate is 97.29%. Meanwhile, a part of participants was investigated twice (one-week interval, *n* = 36) for test–retest analysis. A total of 1,424 subjects were analyzed after excluding participants with missing information. All parents or guardians provided signed informed consent forms, and the study protocols were approved by Ethics Committee of Affiliated Hospital of Jiaxing University (The First Hospital of Jiaxing) (LS2019-107).

### Questionnaire

The questionnaire and correlates of preschoolers’ ST were designed based on previous studies[10–13]. Hard copies were distributed by trained teachers in the classroom and brought home by the children for their parents (mother or/and father) to fill out with pen. The contents of the questionnaire included: (1) children’s information, including gender, date of birth, home-based ST per day (“In generally, how much time for your children to spend on screens at home every day, such as watching TV, playing mobile phone/tablet/computer games”), screen accessibility (“How easy it is for preschoolers to touch the screens of televisions, computers, mobile phones, or tablets”), and the number of meals eaten in front of a screen; (2) parents’ information, including education level, home-based ST per day, parent–child screen-viewing behavior, parental perception of child ST (“Children spending too much time in front of screens is unhealthy”); and (3) the family rules regarding child ST (“There are clear rules for children’s behavior in front of a screen such as strict control of the duration of watching animation”). The options used for the five-point Likert scale included “strongly agree”, “agree”, “equals”, “disagree”, and “strongly disagree”. The weighted kappa coefficients of the ST questions based on our test–retest analysis for preschoolers, fathers, and mothers were 0.76, 0.69, and 0.72, respectively.

### Statistical analysis

Categories were expressed as percentages, and the chi-square test was used for comparison between groups. Continuous variables were expressed as the mean and standard deviation, and an independent sample t-test was used for comparison between groups. A multivariate logistic regression model was used to analyze the factors influencing excessive ST in preschoolers. The odds ratio (OR) and a 95% confidence interval were used to describe the degree of influence of each factor on home-based ST and used an analysis of variance to test the trend. We used SPSS software (version 20.0) for statistical analysis. The significance level was *P* < 0.05 (two-side).

## Results

### Characteristics of preschoolers’ home-based ST

There were 624 (43.8%) preschoolers with a home-based ST of > 1 h/day; among them, 314 (50.3%) were boys and 310 (49.7%) were girls. The gender difference was not significant (*P* > 0.05), as shown in Table 1. The average age of preschoolers whose home-based ST was > 1 h/day was greater than those whose home-based ST was ≤ 1 h/day (*P* < 0.05). The easier it was for preschoolers to touch an electronic screen at home, and the more time they spend eating while viewing the screen, the longer their ST was at home (*P* < 0.05). There were significant differences between the ST of ≤ 1 h/day and > 1 h/day among preschoolers with different parental ST, fathers’ age and education levels, parent–child ST, daily caregivers, and family rules on ST (*P* < 0.05).

**Table 1:** Characteristics of correlates of screen time in Chinese preschoolers

Variables	Screen time (ST), $\overline{\mathrm{X} }$(SD)/n(%), *n* = 1424			X^2^ or t	*P* value
	Total	≤ 1 h/d, *n* = 800	> 1 h/d, *n* = 624		
Child sex					
Girls	704 (49.4)	394 (49.2)	310 (49.7)	0.03	0.872
Boys	720 (50.6)	406 (50.8)	314 (50.3)		
Child age (years)	4.52 (0.86)	4.45 (0.87)	4.60 (0.84)	-3.24	0.001
Parental age					
Father (≥ 40 years)	137 (9.6)	96 (12.0)	41 (6.6)	11.89	< 0.001
Mother (≥ 40 years)	74 (5.2)	45 (5.6)	29 (4.6)	0.68	0.410
Parental education levels					
Father (high school and more)	1133 (79.6)	621 (77.6)	512 (82.1)	4.22	0.040
Mother (high school and more)	1098 (77.1)	603 (75.4)	495 (79.3)	3.10	0.078
Parental ST					
Father (> 1 h/d)	1080 (75.8)	560 (70.0)	520 (83.3)	34.02	< 0.001
Mother (> 1 h/d)	996 (69.9)	515 (64.4)	481 (77.1)	26.93	< 0.001
Accessibility of screen devices	3.68 (1.03)	3.47 (1.07)	3.95 (0.91)	-8.92	< 0.001
Eats while screen viewing	2.74 (1.06)	2.47 (1.03)	3.09 (1.00)	-11.53	< 0.001
Parent–child screen viewing	2.67 (0.90)	2.54 (0.88)	2.84 (0.89)	-6.49	< 0.001
Child cared by parent	809 (56.8)	484 (60.5)	325 (52.1)	10.12	0.001
Parental perception on child ST	1.43 (0.82)	1.40 (0.82)	1.48 (0.82)	-1.95	0.051
Family rules on child ST	1.63 (0.82)	1.46 (0.74)	1.84 (0.87)	-8.85	< 0.001

*ST* Screen time, *h/d* Hour per day, $\overline{X }$*(SD)* Mean (standardized deviation), *n(%)* Number(percentage)

### Multivariate logistic regression on factors affecting preschoolers’ home-based ST

Taking preschoolers’ home-based ST as the dependent variable (≤ 1 h/day = 0; > 1 h/day = 1), and preschoolers’ gender, age, and the parents’ ST as independent variables in the model (Model 1), child age, parent–child screen behaviors, eating while screen viewing, screen accessibility, daily caregivers, mothers’ ST, parental perception, and family rules for child ST were shown to be significant (*P* < 0.05), as can be seen in Table 2. When the father’s education level, age, and ST were included additionally (Model 2), the fathers’ age and ST were significant (*P* < 0.001), but mothers’ ST was not (*P* > 0.05).

**Table 2:** Multivariate logistic regression models for correlates of ST in Chinese preschoolers

Variables	References	Screen time (ST, ≤ 1 h/d vs. > 1 h/d)			
		Model 1		Model 2	
		OR (95%CI)	*P*	OR (95%CI)	*P*
Child					
Sex	Girls	1.03 (0.82,1.30)	0.787	1.03 (0.82,1.31)	0.782
Age	Younger	1.21 (1.05,1.39)	0.008	1.23 (1.07,1.41)	0.004
Eats while screen viewing	Less	1.58 (1.40,1.77)	< 0.001	1.59 (1.41,1.79)	< 0.001
Screen Accessibility	Poor	1.36 (1.20,1.53)	< 0.001	1.35 (1.19,1.52)	< 0.001
Caregiver (grandparent)	Parent	1.40 (1.10,1.77)	0.006	1.39 (1.09,1.76)	0.007
Parent–child screen viewing	Less	1.24 (1.08,1.42)	0.002	1.22 (1.06,1.40)	0.005
Parental perception on child ST	Harmful	1.14 (0.98,1.32)	0.082	1.16 (1.00,1.35)	0.058
Family rules on child ST	Clear	1.60 (1.38,1.86)	< 0.001	1.63 (1.40,1.88)	< 0.001
Mother					
ST	≤ 1 h/d	1.58 (1.22,2.06)	< 0.001	1.26 (0.92,1.71)	0.147
Education levels	≥ high school	0.91 (0.68,1.22)	0.543	1.10 (0.76,1.58)	0.612
Age	≥ 40 years	1.11 (0.64,1.92)	0.702	0.65 (0.34,1.26)	0.200
Father					
ST	≤ 1 h/d			1.63 (1.16,2.28)	0.005
Education levels	≥ high school			0.87 (0.60,1.27)	0.481
Age	≥ 40 years			2.26 (1.34,3.81)	0.002

*ST* Screen time, *h/d* Hour per day, *OR* Odds ratio, *CI* Confidence interval

To further explore the dose–effect relationship between ST and related factors, the home-based ST of preschoolers was further divided into three levels: ≤ 1 h/day, 1–2 h/day, and > 2 h/day. The results showed that older ages of preschoolers, longer fathers’ ST, easier access to ST, more frequent eating by preschoolers while screen viewing, worse parental perception of child ST, and less clear family rules about ST were associated with longer preschoolers’ home-based ST (*P* < 0.05; Table 3). In addition, the trend relationship (changes in OR) between fathers’ ST and preschoolers’ home-based ST was significant (*P* < 0.001; data not shown).

**Table 3:** Multinomial logistic regression model for screen time in Chinese preschoolers

Variables	References	Screen time (ST)			
		1–2 h/d vs. ≤ 1 h/d		> 2 h/d vs. ≤ 1 h/d	
		OR (95%CI)	*P*	OR (95%CI)	*P*
Child					
Sex	Girls	1.01 (0.78,1.29)	0.969	1.06 (0.74,1.52)	0.753
Age	Younger	1.19 (1.03,1.39)	0.021	1.40 (1.12,1.75)	0.003
Eats while screen viewing	Less	1.52 (1.34,1.73)	< 0.001	1.86 (1.54,2.25)	< 0.001
Screen Accessibility	Poor	1.22 (1.07,1.39)	0.003	1.83 (1.48,2.27)	< 0.001
Caregiver (grandparent)	Parent	1.38 (1.06,1.78)	0.015	1.45 (1.00,2.10)	0.048
Parent–child screen viewing	Less	1.23 (1.06,1.42)	0.006	1.22 (0.99,1.50)	0.061
Father ST					
1–2 h/d	≤ 1 h/d	1.22 (0.84,1.79)	0.297	3.06 (1.53,6.11)	0.002
> 2 h/d	≤ 1 h/d	1.44 (0.95,2.19)	0.083	5.93 (2.88,12.21)	< 0.001
Mother ST					
1–2 h/d	≤ 1 h/d	1.32 (0.92,1.88)	0.127	0.94 (0.54,1.64)	0.819
> 2 h/d	≤ 1 h/d	1.11 (0.74,1.66)	0.627	1.15 (0.64,2.08)	0.637
Father education levels	≥ high school	0.85 (0.57,1.28)	0.432	0.99 (0.56,1.76)	0.974
Mother education levels	< high school	0.95 (0.64,1.42)	0.811	1.70 (0.98,2.95)	0.061
Father age	≥ 40 years	2.17 (1.22,3.83)	0.008	2.27 (0.97,5.34)	0.059
Mother age	≥ 40 years	0.76 (0.37,1.58)	0.460	0.49 (0.18,1.36)	0.171
Parental perception on child ST	Harmful	1.08 (0.91,1.28)	0.375	1.41 (1.14,1.76)	0.002
Family rules on child ST	Clear	1.54 (1.32,1.81)	< 0.001	1.96 (1.58,2.43)	< 0.001

*ST* Screen time, *h/d* Hour per day, *OR* Odds ratio, *CI* Confidence interval

## Discussion

The results of this study show that preschoolers’ ST is closely related to the preschoolers’ age, fathers’ ST, eating while screen viewing, screen accessibility, parents’ perception of child ST, and family ST rules. Meanwhile, the potential adverse impact of the mothers’ ST on preschoolers’ screen viewing was explained mostly by the fathers’ ST in our results. This suggests that reducing parents’ (especially fathers’) ST, improving parental awareness of the potential harmful effects of prolonged home-based ST for preschoolers, formulating family ST rules, and reducing screen accessibility may be of great significance for controlling preschoolers’ ST in China.

The results on the positive correlation between parental ST and preschoolers’ ST are consistent with those of previous related studies [11, 13–15]. Many studies have shown that parents’ ST is closely related to children’s ST. Longer daily ST of parents is a risk factor for children’s ST being > 1 h/day [13, 16]. Studies have also shown that the use of electronic media by preschoolers is easily influenced by family members, and this influence can be exerted in a variety of ways (e.g., children learning from their parents’ screen-viewing behavior) [11, 15]. The theory of social cognition suggests that children may develop their own screen-viewing behavior by observing and learning from that of their parents’. Therefore, reducing the home-based ST of preschoolers should start with the people around them (such as parents, grandparents, or other caregivers), especially to make parents aware of the modeling effect of their behavior on preschoolers. However, studies have shown that some parents appear to be unaware that their screen-viewing behavior is setting standards and imitation targets for preschoolers [5]. Mean while, the family rules of parents regulating screen viewing are related to less preschoolers’ home-based ST [11], which is consistent with the results of the present study. However, more than half of the parents seldom formulate rules related to children’s ST [17, 18]. Therefore, clear family rules of screen-viewing behavior may have a positive influence on reducing the ST of preschoolers at home.

The results of this study show that the relationship between fathers' and mothers' ST and that of their preschool children at home does not appear independently, and that the fathers’ ST seems to have a greater relationship to the preschoolers’ ST based on our cross-sectional data. This may be due to the fact that fathers are often better at using electronic media than mothers and have more knowledge of and more opportunities to be exposed to electronic media, which may have a greater impact on children’s related behavior. Moreover, fathers in China are often less involved in the upbringing of preschoolers. Studies have shown that there are currently unscientific parenting ways (e.g., fathers are often absent in early childhood parenting) and fewer father-son interactions than mother-son interactions, that family education concepts include an insufficient understanding of the unique educational significance of fathers [19]. Therefore, fathers’ own ST and related perceptions need to be highlighted in the prevention and control of Chinese preschoolers’ home-based ST in the future.

To the best of our knowledge, few studies have addressed the prevalence and predictors of preschoolers’ screen exposure at mealtime. R Jusien et al. [20] found that more than half of the preschoolers were exposed to screens at mealtime; this, in addition to the status of preschoolers as only children, increased their likelihood of being fed in front of a screen each day. The results of our study show that the more preschoolers ate in front of the screen, the higher their ST was. This may be related to factors such as appetite promotion (e.g., food advertisements), lack of knowledge of healthy eating among parents, and addiction to home-based screen use leading to cognitive associations between screen use and eating behaviors [20]. Therefore, it is necessary to regulate preschoolers’ eating behaviors in the preschool years; parental modeling and family strategies were essential in this regard [21, 22]. The above mentioned factors prompt considerable concern among pediatricians and child care providers about normative eating behaviors and parental interventions among families for their children’s screen media use, with particular attention to only children. The parents and caregivers of young children should be encouraged to avoid screen use for their children at mealtimes [23]; electronic screens should be placed as far away from the eating areas as possible and alternative behaviors (such as parent–child interaction) should be adopted to reduce ST during meals.

Admittedly, there are some limitations to this study. First, the data in this study were derived from a questionnaire and therefore, may be subjective/biased. Although electronic devices (e.g. video cameras) can record children’s ST more objectively, they are difficult to apply to a large sample survey because of the cost of the equipment and relatively complicated data collection. Second, caution should be used when generalizing our results to other populations (such as rural districts), as the participants of this study were recruited from urban communities of eastern China.

## Conclusions

Parents’ (especially fathers’) ST, family rules regarding child ST, and screen accessibility are important risk factors for excessive home-based preschoolers’ ST in our cross-sectional study, with a dose–effect relationship also having been observed. This suggests that efforts to reduce preschoolers' ST should focus on intervening in parental behavior of screen viewing, formulating family ST rules, reducing screen accessibility, and therefore reducing preschoolers’ ST at home. Further studies using a longitudinal design are required.

## Data Availability

The datasets generated and/or analyzed during the current study are not publicly available due to our data needs to be further analyzed and counted for more publications but are available from the corresponding author on reasonable request.

## Abbreviations

OR: Odds ratio
ST: Screen time
